# The Persistence of Mouse Iso-Antibodies In Vivo

**DOI:** 10.1038/bjc.1955.17

**Published:** 1955-03

**Authors:** D. B. Amos


### THE PERSISTENCE OF MOUSE ISO-ANTIBODIES *IN VIVO*.

D. B. AMOS.

*From the Department of Pathology, Medical School, Guy's Hospital, London, S.E.*1.

Received for publication January 1, 1955.

THE protection that can be demonstrated following the incubation of certain tumour cells with iso-immune sera *in vitro* is in striking contrast with the failure of the same sera to protect passively *in vivo* (see review by Hauschka, 1952), but before these findings can be interpreted it should be shown that the conditions obtaining in the body are as favourable for protection as those produced *in vitro.*

There are a number of factors that might be responsible for the failure of serum to protect, few of which have received sufficient attention. Foremost is the question of the activity of the serum, and of the relationship between this activity and the cell dose. Activity may either be measured in terms of protection or through some non-functional property, such as agglutinating power. The former would be too costly both in time and in animals for regular use, the measurement of the second has only recently been made simple and reliable following the introduction of the Dextran technique (Gorer and Mikulska, 1954). Consequently most of the work has been done on unstandardised serum. Similarly the cell dose has received little attention, although it is well known that a given volume of serum will only protect against a limited number of cells. Many experiments have been performed using a lumpy suspension or even solid fragments of tumour, in which case it is probable that serum cannot penetrate to the centre of the mass before the transplant is established. Under these circumstances it is not surprising that success has not been reported.

The object of this investigation was to provide a possible basis for a future attack on problems of passive immunity to tumours. During the course of the work it became apparent that the method would be useful for detecting antigens in genetical experiments and the second part of the paper is devoted to the results obtained.

### MATERIALS AND METHODS.

The origin of the four pure line strains of mice, A, Bagg albino C (Balb.C), $C_3H$ and C57 Black, together with a description of the C57 Black leukosis E.L.4, the production of antibodies, and details of the Dextran method of haemagglutination have already been published (Gorer and Mikulska, 1954).

After a few preliminary experiments in which the intravenous route was used, all antibodies were injected into the peritoneum. 0·1 ml. of serum was introduced into the peritoneal cavity through a fine-bore needle, care being taken to avoid leaking back. Equilibrium was reached in the bloodstream within an hour in homologous animals. When blood was required for testing, the animals were warmed to 37° C. before being anaesthetised with ether and bled from the tail. It was found that enough blood for titration could always be obtained from a single mouse. The serum was removed from the clot and titrated immediately or stored in ampoules at $-20°$ C. for not more than 3 days. In some experiments designed to test for the persistence of antibodies, and in the genetical experiments, a single individual was bled ; in the others, groups of three were bled simultaneously and the serum either pooled or titrated individually.

*Backcross mice.*—A backcross called the C backcross was obtained by mating Balb.C with C57 Black, and crossing the resultant $F_1$ with Balb.C.

*Protection.*—The simple technique already described (Gorer, 1942) has been followed. Fresh leukotic cells from ascitic fluid were suspended in normal mouse serum at a concentration of about one million cells in 0·1 ml. From this stock suspension a series of dilutions in normal serum was made covering the range required. An equal volume of immune serum was added and the mixture allowed to stand on the bench for 15–20 minutes and then inoculated subcutaneously into susceptible mice.

### RESULTS.

*Persistence of antibodies against E.L.4.*

The antibodies studied here are those developed against the hist-compatibility system known as H-2 (Gorer, Lyman and Snell, 1948), which appears to be similar to the Rh system in man. Individual components are often shared between different strains, so that antibodies against E.L.4. produced in one strain will usually also agglutinate cells of other strains (Amos, 1953; Gorer and Mikulska, 1954; Hoecker, Counce and Smith, 1954) ; and antibodies may be detected by the use of several different red cells. In general, A strain cells are the most readily agglutinated and provide the most sensitive indicator of the presence of antibody.

After antibodies have been passaged through the homologous strain their agglutination reactions are similar to those of the original serum, but of lower titre owing to some degree of dilution. Thus Balb.C. anti E.L.4 may have an initial titre of 1/4000 for A cells and 1/2000 for C57Bl cells. After passage for 24 hours in Balb.C these values would be in the region of 1/256 for A cells and 1/128 for C57Bl., the titres falling slowly during the next 10 days to 1/16–1/32 and 1/4–1/16 respectively. Two such die-away curves are shown in Fig. 1. Here Balb.C anti E.L.4 has been injected into Balb.C, and $C_3H$ anti E.L.4 into $C_3H$ mice, the animals have been bled at intervals and the titres of circulating antibodies estimated against C57Bl. cells. For Balb.C anti E.L.4 the titre after 24 hours was 1/256 this level slowly falling to 1/16 after 8 days, the $C_3H$ anti E.L.4 was a less active serum and from an initial level of 1/64 fell to 1/8 after the same interval.

The titre of circulating antibody after 18 hours is proportional to the amount of serum injected, for a range studied from 0·01 ml. to 0·2 ml. For 0·1 ml. of serum the dilution appears to be about 1/20. Assuming the blood volume to be 63·2 c.c. per kilo (Oakley and Warrack, 1940) and the haematocrit 43 per cent (Wintrobe, 1933) for mice of about 25 g. in weight, the serum volume would be approximately 0·7 ml. and would account for a dilution of about 1/8, so that rather more than half of the serum is being lost from the circulation, probably into the lymphatic and extracellular fluids.

In six separate experiments lasting from 8 to 12 days haemagglutinins prepared against E.L.4 have been found to persist for a considerable time, and although there was too much variation between different samples of antibody for an accurate assessment of half-life, this was of the order of the value given by other workers (Dixon *et al.*, 1952). In some of the experiments there tended to be an initial period of up to 8 days in which the titre remained constant and then fell at about the usual rate. This lag was most noticeable when large amounts or exceptionally high titre sera were used, and was most marked with A strain red cells. Haemagglutination does not provide a good indicator for estimating half-life, but is a valuable method for detecting the continuing presence of antibody.

FIG. 1.—The persistence of antibodies against E.L.4 in the homologous strain ; titrated against C57Bl. cells.

Protective antibodies also survive passage through the homologous strain, 0·2 of Balb.C anti E.L.4 was inoculated into some Balb.C mice which were bled out after 2 days. A protection experiment was then set up using 20,000 leukotic cells with 0·1 ml. of passaged antibody in the experimental group and 10,000 and 20,000 cells in normal serum for the controls. The control mice all developed tumours by the 13th day whilst 0·1 ml. of passaged antibody gave complete protection for 34 days, when the experiment was discontinued. A similar experiment in which a different serum was passaged for 3 days gave the same result.

The circulating antibody can also be absorbed by free leukaemic cells, but apparently not by cells outside the circulation. Three groups of Balb.C mice were taken, the control was given 0·1 ml. Balb.C anti E.L.4 alone, the first experimental group was given the same dose of antibody intraperitoneally followed almost immediately by 0·2 ml. of packed E.L.4 cells intravenously, the second was given the same dose of E.L.4 intravenously but the serum was only given after 18 hours. Serum from the first group gave a titre of only 1/4 against C57Bl. cells, whilst for the controls and the second experimental group the titres were 1/32–1/64. Another experiment along similar lines was performed when homologous antibody was given to a group of six Balb.C mice that had actively growing tumours of E.L.4. The course of the transplanted leukosis in Balb.C is of progressive growth up to 7–8 days followed by rapid regression at about the 10th day, antibodies are not detectable in the blood until the tumour begins to regress. In this case the antibody titre in the animals with growing tumours followed closely the titre in a control group without tumours until the 9th day, when there was an abrupt rise in titre in the experimental group, so that whilst E.L.4 will absorb antibodies when freely circulating in the blood stream, it is unable to do so when established in the tissues. This failure to absorb by the growing tumours cannot be ascribed to saturation with locally produced antibody, as similar tumours absorb quite readily *in vitro*, but must be due to lack of intimate contact between the antibody and leukaemic cells. It can readily be seen that the use of tumour fragments could lead to the same result, probably purely a mechanical effect.

*In vivo absorption.*—In contradistinction to their prolonged survival in the homologous strain, passively transferred antibodies are quickly removed in the presence of the corresponding antigen. When 0·1 ml. of Balb.C anti E.L.4 was given intravenously to C57Bl. the titre against A cells after 5 minutes had fallen to 1/16, after 15 minutes to 1/4 and after 30 minutes could no longer be detected, the titre in Balb.C given the same dose remaining at 1/256. Even when the relatively enormous dose of 0·5 ml. of the same serum was given intravenously to the blacks the level after one hour was only 1/4, and after two hours the agglutinating antibodies had been completely absorbed.

This degree of absorption could not have been achieved by the red cells alone. Details of the antigenic structure of E.L.4 will be given by Gorer and Mikulska in a later paper, so that here it is sufficient to mention two of the antigens involved. The antigenic component H2–B is present in E.L.4 and in the tissues and red cells of C57Bl., H2–E is present in E.L.4 and the fixed tissues of C57Bl. is doubtfully present on the red cells ; it is however shared by other strains and is present on the red cells of the A strain. After repeated absorption with C57Bl. red cells Balb.C anti E.L.4 will still agglutinate A strain red cells, C57Bl. liver and E.L.4 will remove all the agglutinins. The removal of the antibodies by the blacks *in vivo* must therefore be due to contact with other cells, probably those of the bone-marrow and possibly by the cells of the liver and splenic sinusoids, etc.

TABLE I.—*Comparison between Absorption with Liver and* in vivo *Absorption of Antibodies against E.L.4.*

| Antibody. | Tested on. | Titre* unabsorbed. | Titre* after absorption with liver from: A. | Balb.C. | Titre* after passage through: A. | Balb.C. |
|---|---|---|---|---|---|---|
| Balb.C anti-E.L.4 | A cells | Over 2000 | 0 | 1024 | 0 | 256 |
| | C57Bl. cells | Over 2000 | 256 | 1024 | 32–64 | 64 |
| $C_3H$ anti-E.L.4 | A cells | 256–2048 | 0 | 0 | 0 | 0 |
| | C57Bl. cells | 512–4000 | 256–512 | 256–512 | 64 | 32–64 |

* Titres expressed as reciprocals.

A comparison of some results obtained by *in vivo* and *in vitro* absorption is given in Table I. For the antibody Balb.C anti E.L.4, A strain liver removes all the antibodies against its own cells and lowers the titre against C57Bl. cells from over 1/2,000 to 1/256, a control absorption with Balb.C liver lowers the titre against both cells slightly. Figures for the *in vivo* absorption are similar, but the titres are lower. A similar situation occurs with $C_3H$ anti E.L.4 where both A and Balb.C remove the agglutinins against A cells but not against C57Bl. Although there has been a considerable degree of dilution in the *in vivo* series the results are clear cut and there does not appear to have been any untoward degree of non-specific absorption. There is little point in using the technique to study the absorptive capacities of pure line strains, as liver is readily available ; but in a genetical experiment the individual mouse may be valuable either for use in further experiments or for breeding. Any number of *in vivo* absorptions can be carried out provided they are adequately spaced.

TABLE II.—*Serological Typing of C backcross mice by direct Agglutination and by* in vivo *Absorption.*

| Mouse No. | Direct agglutination : Balb.C anti-E.L.4. | A anti-E.L.4. | *In vivo* absorbed serum tested on : A cells Balb.C anti-E.L.4. | C57Bl. cells A anti-E.L.4. |
|---|---|---|---|---|
| 17 | + | – | + | + |
| 20 | + | + | – | – |
| 21 | + | + | – | – |
| 23 | – | – | + | + |
| 29 | + | + | – | – |
| 36 | – | – | + | + |

In any serological typing of a genetical experiment there are always a few animals giving weak reactions. Some of these may be due to the serum used containing antibodies against subsidiary antigens (Amos, Gorer, Mikulska, 1955), simply repeating the direct agglutination with a different serum may serve to distinguish these. The classification of the others can only be decided by absorption. Red cell absorption is impracticable for removing " Dextran " antibodies, so that *in vivo* absorption is the method of choice. Table II shows some results of such an absorption with C backcross mice. The columns on the left show the results of direct agglutination with Balb.C anti E.L.4 and A anti E.L.4, the former detecting the presence of the antigens H2-B and H2-E, the latter H2-B. On the right is shown the result of testing the same two sera after passage through the backcross mice. Where the antigen is shown to be present on direct agglutination the corresponding antigen is removed by *in vivo* absorption. Mouse 17 gave a positive result with Balb.C anti-E.L.4 but not A anti-E.L.4 on direct testing, and this result was again obtained when the mouse was tested with a different serum. *In vivo* absorption showed that the H-2 antibodies were not absorbed, the reaction was due to some subsidiary antibody.

Although the technique is very straightforward, there are a few precautions that must be taken. Different sera must not be given at intervals of less than 3–4 weeks in order to allow the residual antibodies to decay and to allow the animal to make good the blood loss. Because animals used in a genetical experiment may have less of the antigen than the pure line it is advisable to allow 18

hours to elapse before bleeding. Finally, it is important to avoid sera from mice that have been too strongly immunised, as these may contain subsidiary antibodies which may give rise to difficulty in the interpretation of results.

### DISCUSSION.

Gorer (1942) suggested that haemagglutinins and protective antibodies were distinct entities but this conclusion was based upon results with saline agglutination, which is an inefficient method of detecting antibodies and at that time the extreme complexity of the H-2 system was unknown. However, the statement may well be correct. Even if that is so, it is possible that the stability of protective antibody may be similar to that of the haemagglutinins. Experiments with Dr. Gorer indicate that their stability *in vitro* is similar and the protection experiments reported above indicate that this is also true *in vivo*. It would seem therefore that the decay of antibody is not too rapid to render passive immunisation impossible, but it would be unreasonable to expect success with cell dosages that are far beyond the power of a serum to inhibit when mixed *in vitro* prior to inoculation.

### SUMMARY AND CONCLUSIONS.

When antibodies against a C57 Black leukosis E.L.4 are inoculated into non-immune homologous mice they may be detected in the bloodstream for as long as 12 days. Variations are too great for an estimate of the half-life to be made, but this appeared to be at least two days and in some cases longer.

Protective antibodies can also be detected in the blood of similarly treated animals for at least 3 days.

Passage through backcross mice results in absorption of antibodies by animals having the corresponding antigen.

My thanks are due to Dr. P. A. Gorer for helpful criticism and advice and to Mrs. Z. B. Mikulska for titrating some of the sera.

### REFERENCES.

AMOS, D. B.—(1953) *Brit. J. exp. Path.*, **34**, 464.

*Idem*, GORER, P. A. AND MIKULSKA, Z. B.—(1955) *Brit. J. Cancer*, **9**, 209.

DIXON, F. J., TALMAGE, P., MAURER, P. H. AND DEICHMILLER, MARIA—(1952) *J. exp. Med.*, **96**, 313.

GORER, P. A.—(1942) *J. Path. Bact.*, **54**, 51.

*Idem*, LYMAN, S. AND SNELL, G. D.—(1948) *Proc. Roy. Soc.* B., **135**, 499.

*Idem* AND MIKULSKA, Z. B.—(1954) *Cancer Res.*, **14**, 651.

HAUSCHKA, T. S.—(1952) *Ibid.*, **12**, 615.

HOECKER, G. S., COUNCE, S. J. AND SMITH, P.—(1954) *Proc. nat. Acad. Sci., Wash.*, **40**, 1040.

OAKLEY, C. L. AND WARRACK, G. HARRIET.—(1940) *J. Path. Bact.*, **50**, 372.

WINTROBE, M. M.—(1933) *Folia haemat. Lpz.*, **51**, 32.
1040.


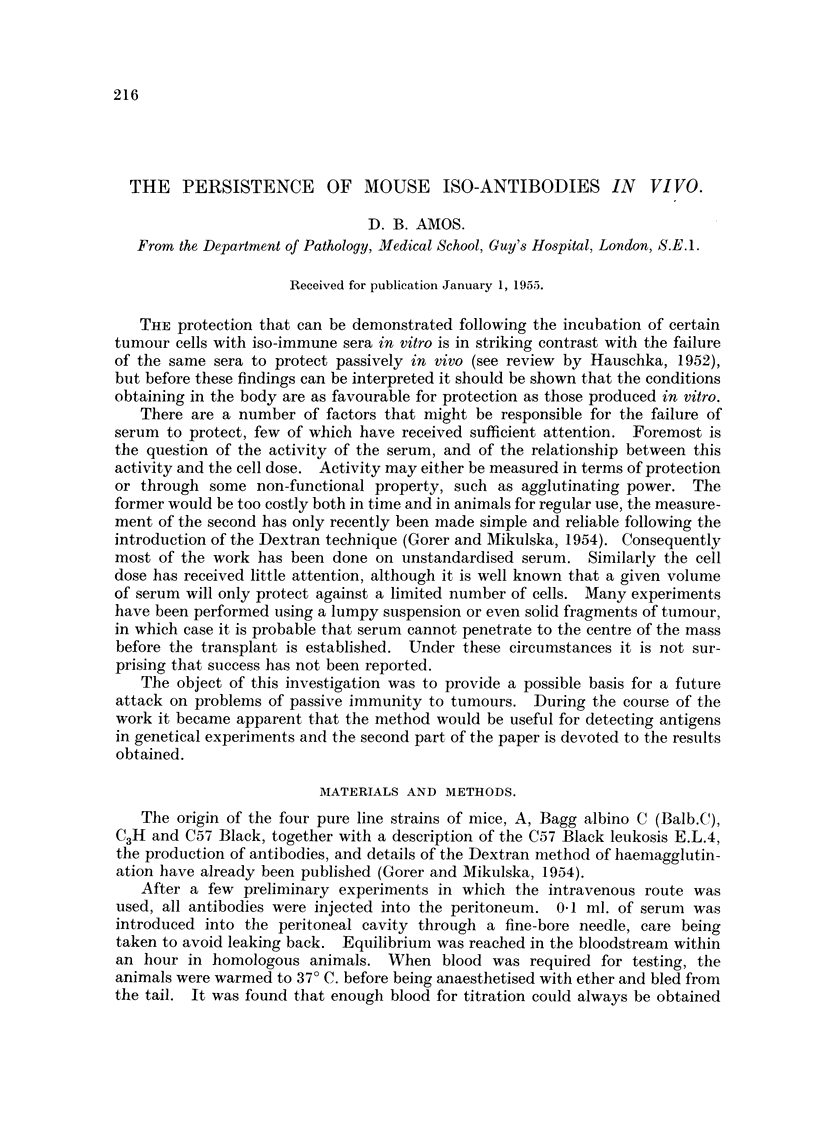

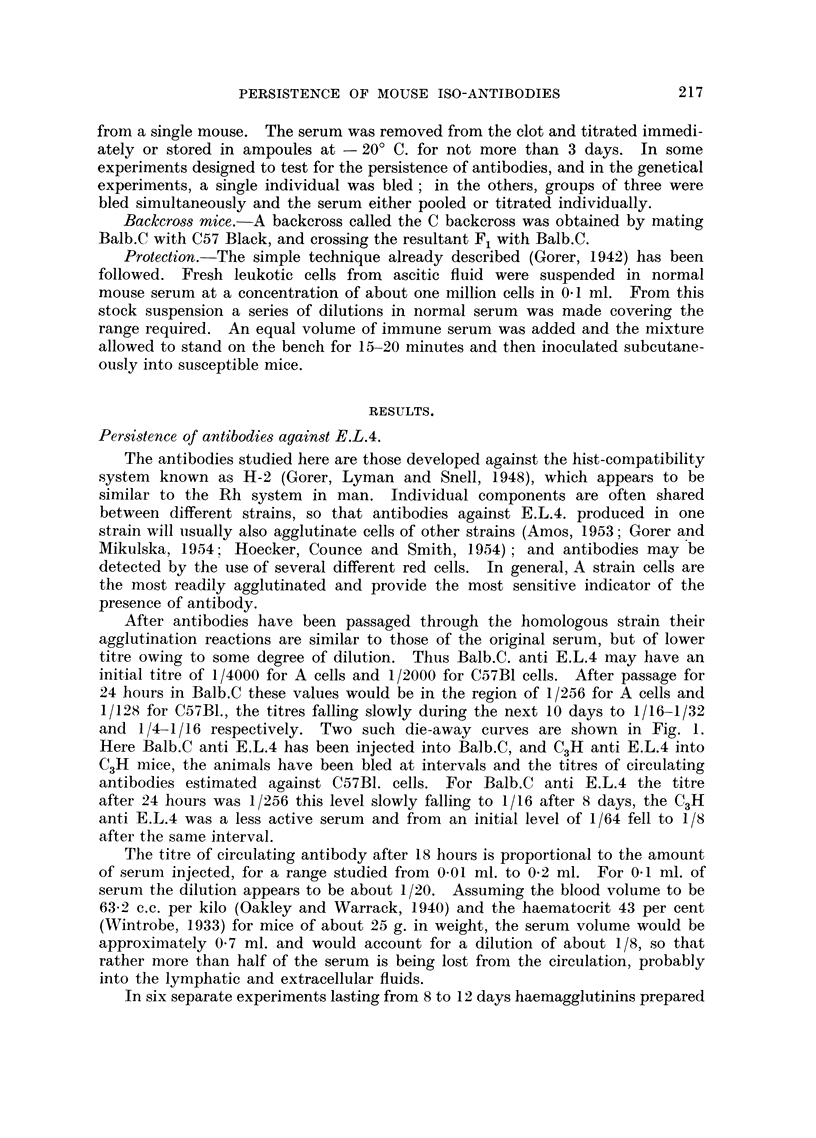

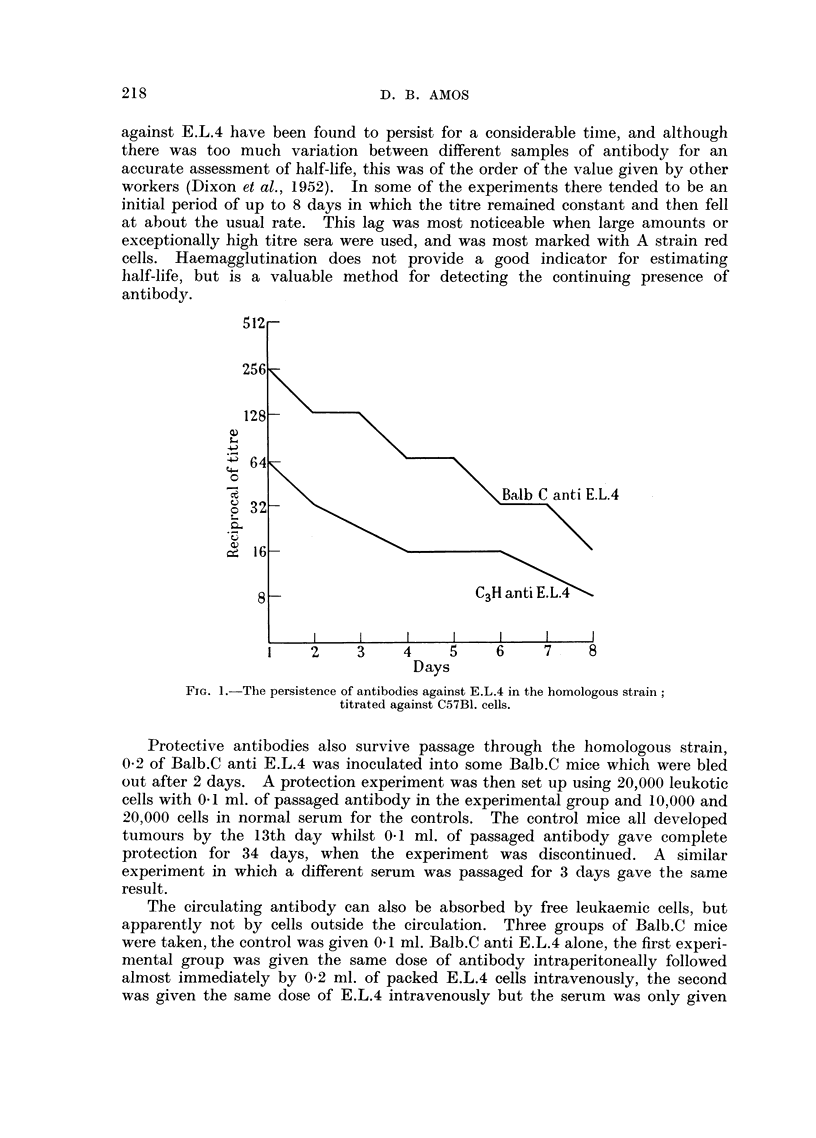

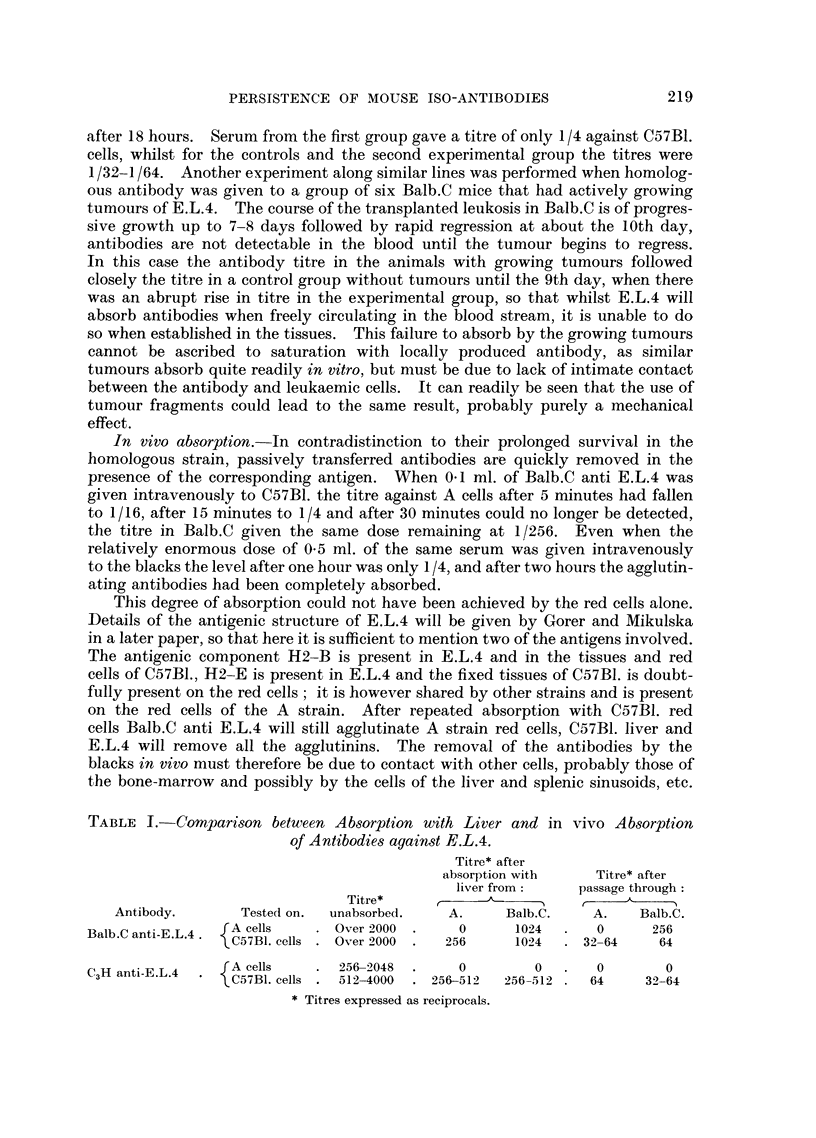

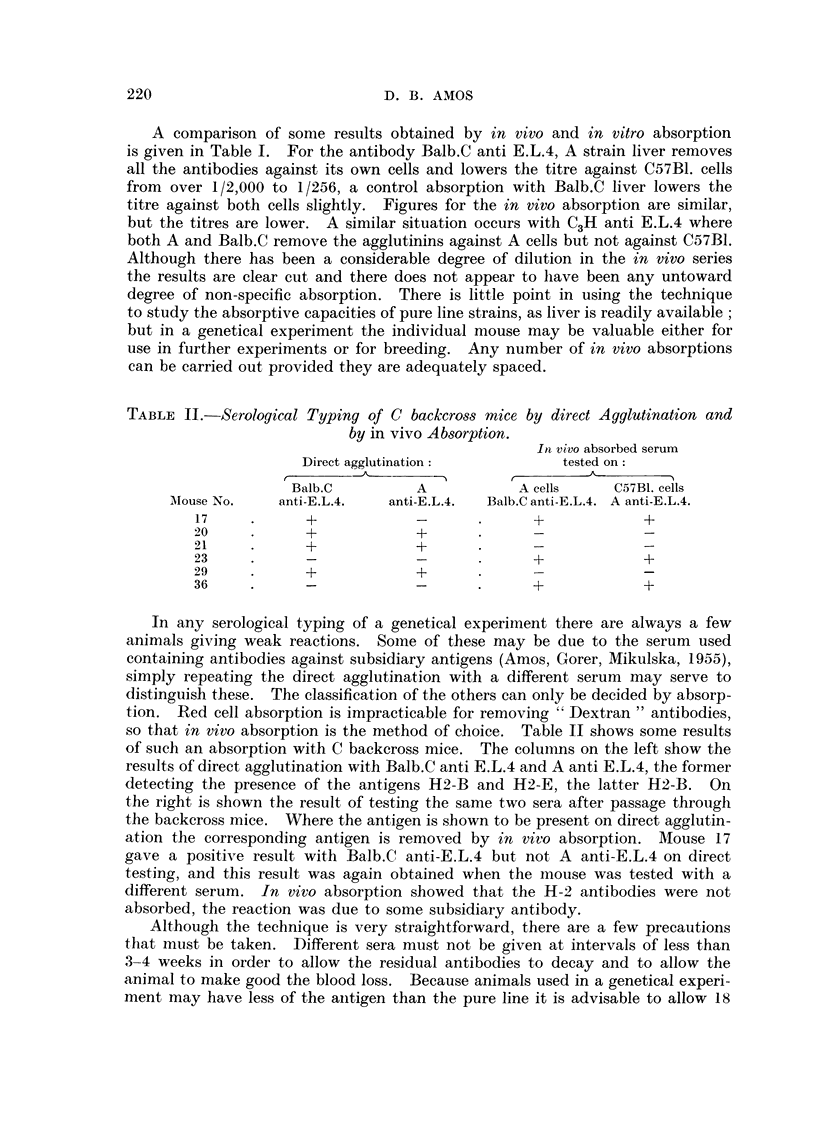

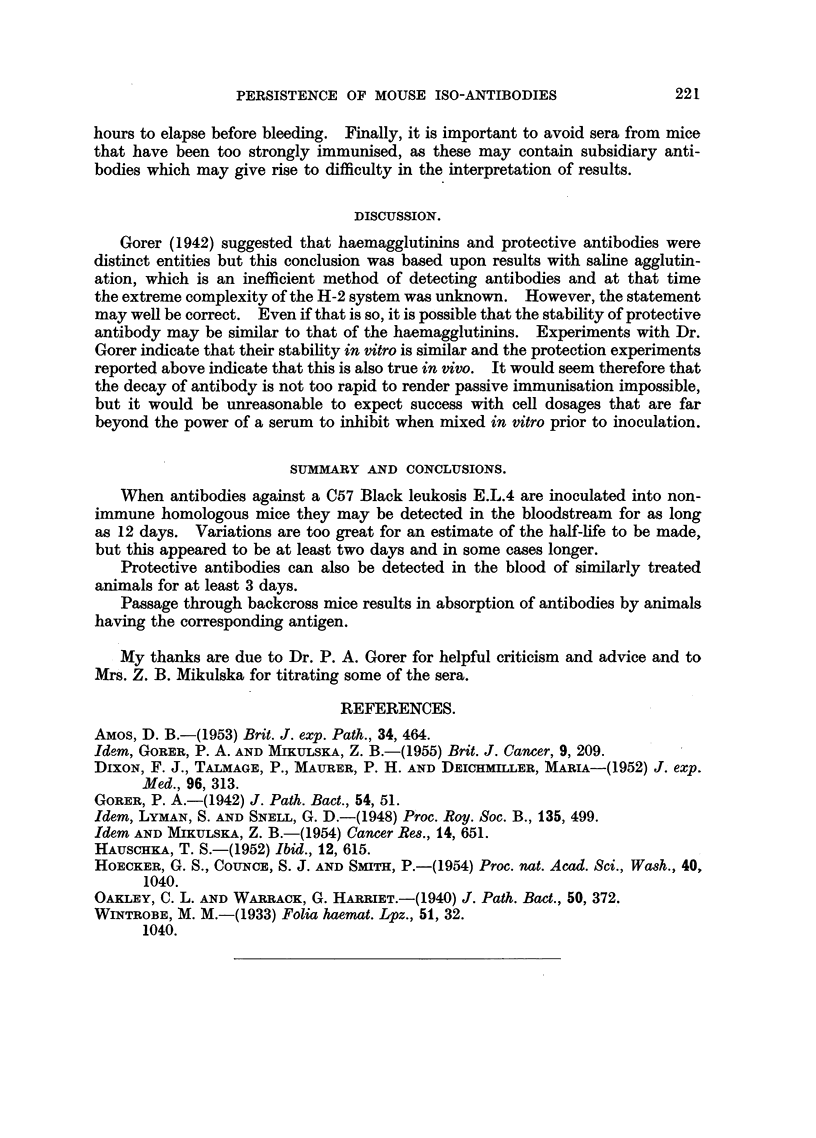


## References

[OCR_00343] DIXON F. J., TALMAGE D. W., MAURER P. H., DEICHMILLER M. (1952). The half-life on homologous gamma globulin (antibody) in several species.. J Exp Med.

